# Association of Gestational Diabetes Mellitus with Adverse Pregnancy Outcomes and Its Interaction with Maternal Age in Chinese Urban Women

**DOI:** 10.1155/2021/5516937

**Published:** 2021-05-18

**Authors:** Xueyin Wang, Xiaosong Zhang, Min Zhou, Juan Juan, Xu Wang

**Affiliations:** Department of Obstetrics and Gynecology, Peking University First Hospital, Beijing 10034, China

## Abstract

**Background:**

The prevalence of gestational diabetes mellitus (GDM) has been dramatically increasing worldwide. The aims of this study were to examine associations of GDM with pregnancy outcomes in Chinese urban women and to evaluate the interaction between GDM and other major risk factors for the risk of adverse pregnancy outcomes.

**Methods:**

A retrospective analysis included 8844 women who delivered live singletons at ≥28 weeks of gestation between June 2012 and March 2013 among Chinese urban women. Structured questionnaires were used to collect information on demographic characteristics, lifestyle behavior, medical history, and pregnancy outcomes. The diagnosis of GDM was made between 24 and 28 gestational weeks according to the International Association of Diabetes and Pregnancy Study Groups criteria. Logistic regression models were used to assess the association of GDM with pregnancy outcomes and to examine the interaction between GDM and other major risk factors including maternal age, prepregnancy body mass index, and gestational weight gain for the risk of pregnancy outcomes.

**Results:**

13.9% of women were diagnosed with GDM. We found that GDM was associated with higher risk of cesarean delivery (odds ratio (OR) = 1.69, 95% CI (confidence interval): 1.48-1.92), preterm birth (OR = 1.32, 95% CI: 1.07-1.64), macrosomia (OR = 1.69, 95% CI: 1.34-2.13), and large for gestational age (LGA, OR = 1.43, 95% CI: 1.18-1.73) after adjustment for potential confounders. We also observed the interaction between GDM and maternal age for the risk of cesarean delivery (*P* for interaction = 0.025), and the OR of GDM for cesarean delivery was 1.71 (95% CI: 1.49-1.97) among women aged less than 35 years.

**Conclusions:**

GDM was associated with an increased risk of cesarean delivery, preterm birth, macrosomia, and LGA in Chinese urban women, and there was an interaction between GDM and maternal age for the risk of cesarean delivery.

## 1. Introduction

GDM is a medical condition of hyperglycemia or glucose intolerance with the first diagnosis in the second or third trimester of pregnancy that is not clearly overt disease prior to gestation [1]. GDM has become a major health concern in pregnant women, and its prevalence has been dramatically increasing worldwide. According to the data from the International Diabetes Federation, GDM was estimated to affect 13.2% of live births in 2019 [2], while the prevalence of GDM ranges from 9.3% to 19.7% in different areas of China and the societal economic burden of GDM was $5.59 billion in 2015 at the Chinese national level [3–6]. GDM has been associated with a number of short- and long-term perinatal outcomes not only for the mothers but also for their offspring. For the mothers, GDM is associated with adverse pregnancy complications and outcomes including pregnancy-induced hypertension (PIH), preeclampsia, and cesarean delivery, as well as the lifetime risk of type 2 diabetes and metabolic syndrome [7–9]. For their offspring, it may increase the risk of macrosomia, large for gestational age (LGA), birth injury, neonatal hypoglycemia, and diabetes and subsequently diabetes and obesity later in life [8, 9].

As major risk factors for GDM, maternal age, prepregnancy body mass index (BMI), and weight gain during pregnancy were also found to be associated with the risk of adverse maternal and infant outcomes [10]. The average age at childbearing has been rising steadily with the fertility rate nearly doubling among women aged above 35 years in recent decades, and women giving birth at advanced maternal age have higher risks of a range of pregnancy complications such as gestational hypertensive disorders, GDM, postpartum haemorrhage, cesarean delivery, and preterm birth [11, 12]. Apart from maternal age, prepregnancy overweight and obesity, affecting 6~24% of Chinese women of childbearing age, have an important influence on both GDM and other maternal and neonatal outcomes such as cesarean delivery, GDM, PIH, neonatal adiposity, and low Apgar scores [10, 13, 14]. Our previous research also reported the associations between prepregnancy overweight and obesity and higher risk of macrosomia and LGA [10]. Furthermore, excessive gestational weight gain (GWG) may contribute to increased risk of cesarean delivery, macrosomia, LGA, hypertensive diseases of pregnancy, and childhood obesity for the offspring, whereas low GWG has been associated with elevated rates of SGA, low birth weight, and preterm birth [15, 16]. Although several studies examined the association of the forementioned risk factors with adverse perinatal outcomes, little is known about the interactive effect between GDM and other risk factors on adverse pregnancy outcomes in urban Chinese women.

Therefore, the objective of the present study was to evaluate associations of GDM with adverse pregnancy outcomes in Chinese urban women and further assess the associations stratified by maternal age, prepregnancy BMI, and GWG categories, and we also examined the interaction between GDM and maternal age and prepregnancy BMI and GWG for the risk of adverse pregnancy outcomes, respectively.

## 2. Materials and Methods

### 2.1. Study Design and Participants

This multicenter, retrospective cohort study of postpartum women was conducted in 14 clinical centers located in urban areas of China from June 2012 to March 2013. Details of this study have been described elsewhere [10]. Briefly, we recruited postpartum women aged 18 years or above with a gestational age of 28-42 weeks and who gave live birth during the 10^th^-19^th^ of the last month of every quarter in order to control for seasonal variations. Among 9152 participants with full medical records, 308 women with pregestational diabetes (*n* = 37), multiple gestation (*n* = 222), pregestational diabetes and multiple gestation (*n* = 1), and preconception history of severe heart disease or chronic renal disease (*n* = 48) were excluded. Overall, the present analysis was restricted to 8844 deliveries. This study was approved by the institutional review board of Peking University First Hospital, and all participants provided written informed consent.

### 2.2. Data Collection

Information on demographic characteristics, lifestyle behavior, medical history of pregnancy, and pregnancy outcomes was collected by using a structured questionnaire after delivery. In-person interviews were conducted to collect information on demographic characteristics such as age, education, employment, and annual household income, as well as lifestyle behavior including drinking and passive smoking during pregnancy, and clinical data such as medical history and pregnancy outcomes was extracted from the medical records.

Gestational age at delivery was determined from the date of the last menstrual period to the date of delivery and expressed in the week after the last menstrual period. If the date was uncertain, ultrasonography was used to determine gestational age. Weight and height at the first antenatal visit prior to the 13^th^ gestational week and weight at the last antenatal visit within 2 weeks before delivery or the time of delivery were extracted from the medical records. Prepregnancy BMI was calculated as the weight in kilograms divided by the square of height measured in meters and classified into three groups according to the Chinese standard [17]: underweight (BMI < 18.5 kg/m^2^), normal weight (18.5 kg/m^2^ ≤ BMI < 24 kg/m^2^), and overweight and obese (BMI ≥ 24 kg/m^2^). The GWG was calculated by subtracting the weight measured at the first antenatal visit from the final weight measured at the last antenatal visit or the time of delivery. All participants were divided into three groups according to GWG, defined by the 25^th^ and 75^th^ percentile of GWG (12.0 and 19.0 kg): lower (GWG < 12 kg), middle (12 kg ≤ GWG < 19 kg), and higher (≥19 kg).

### 2.3. Diagnosis of GDM

According to the International Association of Diabetes and Pregnancy Study Groups (IADPSG) criteria, the diagnosis of GDM should be made when any one of the 75 g oral glucose tolerance test value met or exceeded 5.1 mmol/L at 0 h, 10.0 mmol/L at 1 h, and 8.5 mmol/L at 2 h when performed between 24 and 28 gestational weeks [18].

### 2.4. Definition of Adverse Pregnancy Outcomes

The main outcomes of the study were cesarean delivery, preterm birth, low birth weight, small for gestational age (SGA), macrosomia, and LGA. Preterm birth was defined as all birth before 37 completed weeks or before 259 completed days since the first day of a woman's last menstrual period [19]. Low birth weight was defined as neonatal birth weight < 2500 g and macrosomia as birth weight ≥ 4000 g, respectively. LGA and SGA were indicated by birth weight less than and greater than the 10^th^ and 90^th^ percentile, respectively, for the same gestational age by sex, according to the Chinese neonatal birth weight curve [20].

### 2.5. Statistical Analysis

Demographic characteristics and pregnancy outcomes were expressed as numbers and frequency distributions for categorical variables or median and interquartile range for continuous variables. To compare between groups, the chi-squared test and Mann-Whitney *U* test were performed for categorical variables and skewed distributed continuous variables, respectively. Logistic regression models were conducted to estimate odds ratios (ORs) and their 95% confidence intervals (CIs) of pregnancy outcomes and GDM and interaction between GDM and maternal age/prepregnancy BMI/GWG groups. Models were adjusted for maternal age (continuous), education (high school and below, college or graduate school), employment (unemployed, employed), annual household income (<10000 RMB, 10000-20000 RMB, and <20000 RMB), drinking during pregnancy (yes, no), passive smoking during pregnancy (yes, no), prepregnancy BMI categories (underweight, normal weight, and overweight/obese), parity (primiparous, multiparous), use of assisted reproductive technology (ART; yes, no), folic acid supplementation (yes, no), gestational age at delivery (continuous, except for the outcome of preterm birth), and GWG categories (lower, middle, and higher). The non-GDM group was used as the reference group. Stratified analyses were performed according to maternal age groups (<35 years, ≥35 years), prepregnancy BMI categories (underweight, normal weight, and overweight/obese), and GWG categories (lower, middle, and higher). Analyses were carried out using SAS software version 9.2 (SAS Institute, Cary, NC). All *P* values are two-sided, and statistical significance was defined as *P* < 0.05.

## 3. Results

### 3.1. Participant Characteristics

Among 8844 postpartum women, 1229 (13.9%) were diagnosed with GDM. The average age of study participants was 28.7 years. Distribution of GDM status by study centers is shown in Table S1.  Table 1 shows demographic characteristics of participants according to GDM status. Women with GDM had a higher likelihood of advanced maternal age (≥35 years), prepregnancy overweight and obesity, and use of ART than non-GDM counterparts (all *P* < 0.05). Compared to those unaffected by GDM, women with GDM were more likely to be exposed to passive smoking during pregnancy and less likely to drink during pregnancy and to be in the middle group of GWG (all *P* < 0.05).

### 3.2. Prevalence of Pregnancy Outcomes by GDM

**Figure 1:**
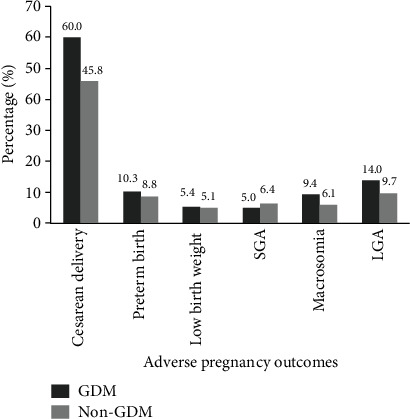
Percentage of adverse pregnancy outcomes by gestational diabetes mellitus. Abbreviations: GDM: gestational diabetes mellitus; SGA: small for gestational age; LGA: large for gestational age.

Figure 1 and Table S2 illustrate the prevalence of adverse pregnancy outcomes by GDM status. Women with GDM had a higher likelihood of cesarean delivery (60.0% vs. 45.8%), macrosomia (9.4% vs. 6.1%), and LGA (14.0% vs. 9.7%) than those who did not have GDM (all *P* < 0.001). However, the prevalence of preterm birth, low birth weight, and SGA was comparable between the two groups (all *P* > 0.05).

### 3.3. Associations of GDM with Pregnancy Outcomes

Table 2 shows associations of adverse pregnancy outcomes with GDM status. After adjustment for potential confounders, GDM was associated with a higher risk for cesarean delivery (OR = 1.69, 95% CI: 1.48-1.92), preterm birth (OR = 1.32, 95% CI: 1.07-1.64), macrosomia (OR = 1.69, 95% CI: 1.34-2.13), and LGA (OR = 1.43, 95% CI: 1.18-1.73).

### 3.4. The Effect of the Interaction between GDM and Maternal Age on Pregnancy Outcomes

**Figure 2:**
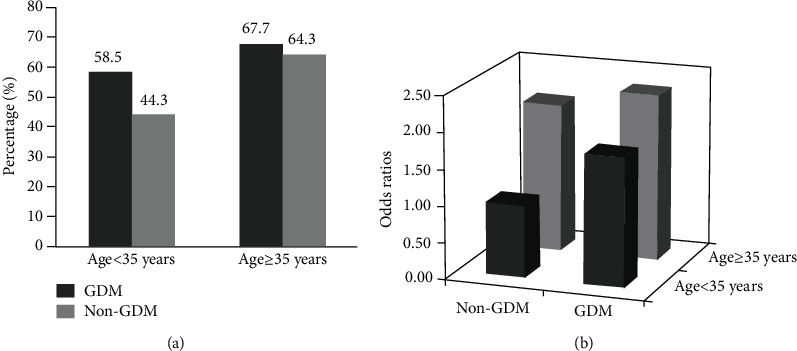
Associations of gestational diabetes mellitus and maternal age with the risk of cesarean delivery. (a) Percentage of gestational diabetes mellitus by maternal age groups. Values are *n* (%). (b) Adjusted odds ratios for cesarean delivery according to different gestational diabetes mellitus and maternal age groups. Values are odds ratios. Adjusted for maternal age, education, employment, annual household income, study centers, drinking during pregnancy, passive smoking during pregnancy, prepregnancy body mass index categories, parity, use of assisted reproductive technology, folic acid supplementation, gestational age at delivery, and gestational weight gain categories. Abbreviations: GDM: gestational diabetes mellitus.

There was a significantly statistical interaction between GDM and maternal age (<35 years, ≥35 years) for the risk of cesarean delivery after adjustment for potential confounders (*P* for interaction = 0.025, Table 3). The prevalence of cesarean delivery was higher in women with GDM than those who did not have GDM regardless of maternal age (age < 35 years: 58.5% vs. 44.3%; age ≥ 35 years: 67.7% vs. 64.3%). Among women of age < 35 years, the OR of GDM for cesarean delivery was in 1.71 (95% CI: 1.49-1.97) after adjustment for potential confounders, while GDM was not significantly associated with the risk of cesarean delivery among those aged 35 years and above. In addition, we further performed the combined analysis to evaluate the effect of GDM and maternal age on the risk of cesarean delivery (Figure 2). Compared to those aged <35 years and having no GDM, women with GDM and aged ≥35 years had a 2.10-fold odds of cesarean delivery (OR = 2.10, 95% CI: 1.48-2.93).

We also examined the relationships between GDM and pregnancy outcomes by prepregnancy BMI and GWG categories, respectively. Results were generally consistent pertaining to associations of GDM with the risk of pregnancy outcomes across different prepregnancy BMI (Table S3) and GWG groups (Table S4).

## 4. Discussion

In this retrospective cohort study of Chinese urban women, 13.9% of women were diagnosed with GDM. We found that GDM was associated with a higher risk of cesarean delivery, preterm birth, macrosomia, and LGA after adjustment for potential confounders. We also observed the interaction between GDM and maternal age for the risk of cesarean delivery.

The positive associations of GDM with increased risk of macrosomia and LGA have been reported in different populations, which is in accordance with our study [21–23]. A retrospective cohort study enrolling more than 21000 Thai women indicated that the rate of macrosomia was significantly higher in the GDM than in the control, and GDM was associated with a 48% increased risk of macrosomia [21]. In addition, the Hyperglycemia and Adverse Pregnancy Outcome (HAPO) study conducted in 15 centers in nine countries demonstrated the continuous positive associations between maternal glucose concentrations and birth weight even among those who had plasma glucose previously considered normal for pregnancy [24]. Previous randomized controlled trials also implied that glycemic control through dietary intervention, physical activity, and/or insulin therapy was able to reduce the risk of macrosomia and LGA [25, 26]. Recently, a prospective cohort study based on a US population suggested that GDM was associated with a larger fetal size that started at gestational week 20 and became significant at gestational week 28, indicating that interventions to mitigate fetal overgrowth related to GDM should be initiated before the diagnosis of GDM [27]. The mechanisms and pathways for the relationship between maternal hyperglycemia and neonatal birth weight are not well described. It might be explained that maternal hyperglycemia and insulin resistance might cause fetal hyperinsulinemia and thus enhanced the utilization of nutrients and consequently leading to fetal overgrowth and adiposity [23, 28].

Another finding of our study is that GDM was associated with an increased risk of preterm birth. Prior research on the relationship between GDM and risk of preterm birth has been still controversial. Similar to our findings, a population study focusing on more than 327,000 singleton pregnancies in Canada indicated that GDM was associated with an increased risk of spontaneous preterm and labor-induced preterm in singleton pregnancies [29]. Moreover, a population-based study conducted in northeastern Germany reported that GDM resulted in a higher percentage of late preterm infants with a gestational age of 32-36 weeks (11.1% vs. 7.0%) [30]. However, several retrospective studies found no increased risk for preterm delivery in women having GDM in comparison to non-GDM counterparts [31, 32]. Therefore, further research is needed to explore the relationship and underlying mechanisms between GDM and the risk of preterm birth.

In addition, we also observed that GDM was associated with a 62% increased risk of cesarean delivery. Consistent with our findings, the data from the Massachusetts Pregnancy to Early Life Longitudinal Data System has shown that women with GDM during one of two sequential pregnancies had an increased risk of cesarean delivery [33]. A large-scale observational national study in France found that the risk of cesarean delivery was increased in women with GDM compared with the nondiabetic population in deliveries after 28 weeks [34]. Another observational study conducted at 15 medical centers in Beijing demonstrated that GDM women had an elevated risk of cesarean delivery compared with normal blood glucose women [35]. The HAPO study revealed that the odds ratio for primary cesarean section increased across categories of maternal glycemia with an OR of 1.86 in the highest category of 1-hour plasma glucose [24]. The association between GDM and the increased risk of cesarean delivery may be influenced by two aspects: on the one hand, maternal hyperglycemia might contribute to fetal overgrowth and large neonatal birth weight, which could lead to the higher risk of cesarean delivery; on the other hand, women developing GDM may affect clinical decision-making due to the higher rates of abnormalities in labor, birth trauma, and fetal distress, which might increase the risk of cesarean delivery [36]. However, potential mechanisms need further exploration in future research.

One interesting finding of our study is that we found a significant interaction between GDM and maternal age in relation to the risk of cesarean delivery. Specifically, we observed that women aged 35 years and above and having GDM had a 2.1-fold increased risk of cesarean delivery compared to those who were under 35 years and did not have GDM. Moreover, the stratified analysis of our study indicated that GDM was much more important in women aged less than 35 years than in those 35 years and above among Chinese urban women. In contrast to our finding, a previous retrospective study focusing on 880 Portuguese women also found the interaction between GDM and maternal age for cesarean delivery by reporting that women older than 35 years presented a higher association between GDM and nonelective cesarean delivery than women younger than 35 years [36]. Differences in findings may be partly due to ethnicity disparities and focus on different types of cesarean delivery. Overall, our finding emphasizes the important public health implications of glycemic control by lifestyle intervention among pregnant women developing GDM, which is particularly crucial in Chinese urban women under 35 years.

The main strengths of our study include a relatively large sample size, using the IADPSG criteria for GDM diagnosis that is reported to be more robust for diagnosis of GDM, and its influence on adverse maternal and perinatal outcomes [37], further assessing the interaction of GDM and other major factors for adverse pregnancy outcomes and adjusting for potential confounding factors comprehensively. However, our study has a few limitations. Firstly, we did not distinguish between emergency and elective cesarean deliveries and did not differentiate spontaneous and indicated preterm birth. Secondly, our data included only the Chinese Han population, and it is unclear whether the results can be extrapolated to women of other ethnic groups. Lastly, we did not collect blood specimens and specific plasma glucose levels such as fasting and 1-hour and 2-hour plasma glucose levels, so we were unable to examine associations of pregnancy outcomes with certain glycemic parameters.

## 5. Conclusions

In summary, our study indicated that GDM was associated with a higher risk of cesarean delivery, preterm birth, macrosomia, and LGA. We also observed the interaction between GDM and maternal age for the risk of cesarean delivery. Our findings emphasize the negative impact of GDM on pregnancy outcomes among Chinese urban women, particularly in those below 35 years old.

## Figures and Tables

**Table 1:** Characteristics of participants by gestational diabetes mellitus.

	All	GDM	Non-GDM	*P* value
Participants, *n* (%)	8844	1229 (13.9)	7615 (86.1)	—
Maternal age (year), median (IQR)	28 (26, 31)	29 (26, 32)	28 (26, 30)	<0.001
Maternal age, *n* (%)				<0.001
<35 years	8095 (91.5)	1074 (87.4)	7021 (92.2)	
≥35 years	749 (8.5)	155 (12.6)	594 (7.8)	
Education, *n* (%)				0.801
High school and below	3346 (37.8)	461 (37.5)	2885 (37.9)	
College or graduate school	5498 (62.2)	768 (62.5)	4730 (62.1)	
Employment, *n* (%)				0.304
Unemployed	6456 (73.0)	912 (74.2)	5544 (72.8)	
Employed	2388 (27.0)	317 (25.8)	2071 (27.2)	
Annual household income (RMB), *n* (%)				0.874
<10000	7071 (80.0)	980 (79.7)	6091 (80.0)	
10001-20000	1288 (14.6)	184 (15.0)	1104 (14.5)	
>20000	485 (5.4)	65 (5.3)	420 (5.5)	
Drinking during pregnancy, *n* (%)				0.014
No	8607 (97.3)	1209 (98.4)	7398 (97.2)	
Yes	237 (2.7)	20 (1.6)	217 (2.8)	
Passive smoking during pregnancy, *n* (%)				0.014
No	7059 (79.8)	949 (77.2)	6110 (80.2)	
Yes	1785 (20.2)	280 (22.8)	1505 (19.8)	
Prepregnancy BMI categories, *n* (%)				<0.001
Underweight	7732 (87.4)	963 (78.4)	6769 (88.9)	
Normal weight	946 (10.7)	204 (16.6)	742 (9.7)	
Overweight/obesity	166 (1.9)	62 (5.0)	104 (1.4)	
Parity, *n* (%)				0.136
Primiparous	7237 (81.8)	987 (80.3)	6250 (82.1)	
Multiparous	1607 (18.2)	242 (19.7)	1365 (17.9)	
Use of ART, *n* (%)				0.020
No	8680 (98.2)	1196 (97.3)	7484 (98.3)	
Yes	164 (1.8)	33 (2.7)	131 (1.7)	
Folic acid supplementation, *n* (%)				0.237
No	4067 (46.0)	546 (44.4)	3521 (46.2)	
Yes	4777 (54.0)	683 (44.6)	4094 (53.8)	
Gestational age at delivery (week), median (IQR)	39 (38, 40)	39 (38, 39)	39 (38, 40)	<0.001
Gestational weight gain (kg)				<0.001
Lower	1282 (14.5)	245 (19.9)	1037 (13.6)	
Middle	6367 (72.0)	833 (67.8)	5534 (72.7)	
Higher	1195 (13.5)	151 (12.3)	1044 (13.7)	

Abbreviations: ART: assisted reproductive technology; BMI: body mass index; GDM: gestational diabetes mellitus. Values are the median (interquartile range) or *n* (%).

**Table 2:** ORs (95% CIs) for pregnancy outcomes by gestational diabetes mellitus.

	Case/noncase	Model 1		Model 2	
		OR (95% CI)	*P* value	OR (95% CI)	*P* value
Cesarean delivery	4227/4617	1.75 (1.54, 1.99)	<0.001	1.69 (1.48, 1.92)	<0.001
Preterm birth^∗^	800/8044	1.37 (1.11, 1.70)	0.004	1.32 (1.07, 1.64)	0.011
Low birth weight	454/8390	1.17 (0.88, 1.56)	0.276	0.86 (0.59, 1.25)	0.422
SGA	548/8296	0.76 (0.57, 1.01)	0.062	0.75 (0.56, 1.01)	0.051
Macrosomia	579/8265	1.58 (1.26, 1.97)	<0.001	1.69 (1.34, 2.13)	<0.001
LGA	908/7936	1.50 (1.24, 1.81)	<0.001	1.43 (1.18, 1.73)	<0.001

Abbreviations: CI: confidence interval; OR: odds ratio; SGA: small for gestational age; LGA: large for gestational age. Model 1 was adjusted for demographic characteristics including maternal age, education, employment, annual household income, and study centers. Model 2 was further adjusted for drinking during pregnancy, passive smoking during pregnancy, prepregnancy body mass index categories, parity, use of assisted reproductive technology, folic acid supplementation, gestational age at delivery, and gestational weight gain categories. Preterm birth was not adjusted for gestational age at delivery^∗^.

**Table 3:** ORs (95% CIs) for pregnancy outcomes by gestational diabetes mellitus, stratified by maternal age.

	Age < 35 years		*Age* ≥ 35 years		*P* for interaction
	OR (95% CI)	*P* value	OR (95% CI)	*P* value	
Cesarean delivery					
Model 1	1.75 (1.53, 2.01)	<0.001	1.27 (0.84, 1.91)	0.255	0.042
Model 2	1.71 (1.49, 1.97)	<0.001	1.13 (0.74, 1.73)	0.559	0.025
Preterm birth^∗^					
Model 1	1.49 (1.19, 1.88)	<0.001	0.75 (0.40, 1.41)	0.376	0.158
Model 2	1.45 (1.15, 1.83)	0.002	0.73 (0.38, 1.40)	0.334	0.133
Low birth weight					
Model 1	1.25 (0.92, 1.69)	0.155	0.83 (0.37, 1.89)	0.665	0.539
Model 2	0.81 (0.54, 1.22)	0.319	2.28 (0.55, 9.43)	0.256	0.383
SGA					
Model 1	0.81 (0.60, 1.10)	0.176	0.63 (0.26, 1.51)	0.298	0.532
Model 2	0.80 (0.59, 1.08)	0.143	0.63 (0.25, 1.58)	0.322	0.463
Macrosomia					
Model 1	1.49 (1.18, 1.90)	0.001	1.58 (0.81, 3.08)	0.178	0.830
Model 2	1.65 (1.28, 2.11)	<0.001	1.52 (0.73, 3.16)	0.265	0.954
LGA					
Model 1	1.43 (1.17, 1.75)	<0.001	1.55 (0.89, 2.72)	0.121	0.963
Model 2	1.39 (1.13, 1.70)	<0.001	1.35 (0.75, 2.44)	0.316	0.898

Abbreviations: CI: confidence interval; OR: odds ratio; SGA: small for gestational age; LGA: large for gestational age. Model 1 was adjusted for demographic characteristics including maternal age, education, employment, annual household income, and study centers. Model 2 was further adjusted for drinking during pregnancy, passive smoking during pregnancy, prepregnancy body mass index categories, parity, use of assisted reproductive technology, folic acid supplementation, gestational age at delivery, and gestational weight gain categories. Preterm birth was not adjusted for gestational age at delivery^∗^.

## Data Availability

The data used to support the findings of this study are available from the corresponding author upon request.

## Supplementary Materials

Supplementary Materials Table S1: distribution of gestational diabetes mellitus by study centers. Table S2: pregnancy outcomes by gestational diabetes mellitus. Table S3: adjusted ORs (95% CIs) for pregnancy outcomes by gestational diabetes mellitus, stratified by prepregnancy body mass index. Table S4: adjusted ORs (95% CIs) for pregnancy outcomes by gestational diabetes mellitus, stratified by gestational weight gain.
