# Supplementary material for: Association of Gestational Diabetes Mellitus with Adverse Pregnancy Outcomes and Its Interaction with Maternal Age in Chinese Urban Women
Source: J Diabetes Res. 2021 May 18;2021:5516937. doi: 10.1155/2021/5516937 (PMC8154302; doi:10.1155/2021/5516937)
Supplement: Supplementary Materials — Table S1: distribution of gestational diabetes mellitus by study centers. Table S2: pregnancy outcomes by gestational diabetes mellitus. Table S3: adjusted ORs (95% CIs) for pregnancy outcomes by gestational diabetes mellitus, stratified by prepregnancy body mass index. Table S4: adjusted ORs (95% CIs) for pregnancy outcomes by gestational diabetes mellitus, stratified by gestational weight gain. [file 5516937.f1.docx]

**Supplementary material**

**STable 1.** Distribution of gestational diabetes mellitus by study centers

| Study centers | n | GDM status | |
| --- | --- | --- | --- |
|  |  | Yes | No |
| 1 | 274 | 117 (42.7) | 157 (57.3) |
| 2 | 646 | 180 (27.9) | 466 (72.1) |
| 3 | 817 | 181 (22.1) | 636 (77.9) |
| 4 | 486 | 116 (23.9) | 370 (76.1) |
| 5 | 836 | 60 (7.2) | 776 (92.8) |
| 6 | 663 | 109 (16.4) | 554 (83.6) |
| 7 | 198 | 43 (21.7) | 155 (78.3) |
| 8 | 795 | 40 (5.0) | 755 (95.0) |
| 9 | 1086 | 64 (5.9) | 1022 (94.1) |
| 10 | 1027 | 90 (8.8) | 937 (91.2) |
| 11 | 296 | 48 (16.2) | 248 (83.8) |
| 12 | 429 | 28 (6.5) | 401 (93.5) |
| 13 | 903 | 140 (15.5) | 763 (84.5) |
| 14 | 388 | 13 (3.3) | 375 (96.7) |
| *P* value | - | <0.001 | |

Abbreviations: GDM, gestational diabetes mellitus.

List of 14 study centers: 1=Peking University First Hospital; 2=Shunyi Women’s & Children’s Hospital of Beijing Children’s Hospital; 3=Tongzhou Maternal & Child Health Hospital of Beijing; 4= Maternal and Child Hospital of Guangdong Province; 5= Shenzhen Maternity & Child Healthcare Hospital; 6= Hunan Province Maternal and Child Health Care Hospital; 7=Xiangya Hospital Central South University; 8=Changsha Hospital for Maternal & Child Health Care; 9=Maternal and Child Hospital of Hubei Province; 10=Wuhan Women and Children Care Center; 11=Sichuan Provincial Hospital for Women and Children; 12=Zigong Hospital for Maternal & Child Health Care; 13=Shanxi Provincial Hospital for Women and Children; 14=Baoji Hospital for Maternal & Child Health Care.

STable 2. Pregnancy outcomes by gestational diabetes mellitus

|  | All | GDM status | | *P* value |
| --- | --- | --- | --- | --- |
|  |  | Yes | No |  |
| Cesarean delivery |  |  |  | <0.001 |
| No | 4617 (52.2) | 492 (40.0) | 4125 (54.2) |  |
| Yes | 4227 (47.8) | 737 (60.0) | 3490 (45.8) |  |
| Preterm birth |  |  |  | 0.090 |
| No | 8044 (91.0) | 1102 (89.7) | 6942 (91.2) |  |
| Yes | 800 (9.0) | 127 (10.3) | 673 (8.8) |  |
| Low birth weight |  |  |  | 0.685 |
| No | 8390 (94.9) | 1163 (94.6) | 7227 (94.9) |  |
| Yes | 454 (5.1) | 66 (5.4) | 388 (5.1) |  |
| SGA |  |  |  | 0.053 |
| No | 8296 (93.8) | 1168 (95.0) | 7128 (93.6) |  |
| Yes | 548 (6.2) | 61 (5.0) | 487 (6.4) |  |
| Macrosomia |  |  |  | <0.001 |
| No | 8265 (93.5) | 1114 (90.6) | 7151 (93.9) |  |
| Yes | 579 (6.5) | 115 (9.4) | 464 (6.1) |  |
| LGA |  |  |  | <0.001 |
| No | 7936 (89.7) | 1057 (86.0) | 6879 (90.3) |  |
| Yes | 908 (10.3) | 172 (14.0) | 736 (9.7) |  |

Abbreviations: CI, confidence interval; GDM, gestational diabetes mellitus; OR, odds ratio; SGA, small-for-gestational age; LGA, large-for-gestational age. Values are n (%).

STable 3. Adjusted ORs (95% CIs) for pregnancy outcomes by gestational diabetes mellitus, stratified by prepregnancy body mass index

|  | Underweight | |  | Normal weight | |  | Overweight/obese | | *P* for interaction |
| --- | --- | --- | --- | --- | --- | --- | --- | --- | --- |
|  | OR (95% CI) | *P* value |  | OR (95% CI) | *P* value |  | OR (95% CI) | *P* value |  |
| Cesarean delivery | 1.72 (1.49, 1.99) | <0.001 |  | 1.49 (1.04, 2.15) | 0.031 |  | 2.11 (0.82, 5.42) | 0.121 | 0.699 |
| Preterm birth^†^ | 1.36 (1.08, 1.73) | 0.010 |  | 1.29 (0.72, 2.31) | 0.386 |  | 3.79 (0.70, 20.69) | 0.124 | 0.605 |
| Low birth weight | 0.84 (0.55, 1.28) | 0.403 |  | 0.96 (0.33, 2.78) | 0.940 |  | 0.27 (0.03, 2.28) | 0.228 | 0.586 |
| SGA | 0.76 (0.56, 1.05) | 0.091 |  | 1.10 (0.50, 2.42) | 0.811 |  | 0.03 (0.01, 1.12) | 0.058 | 0.212 |
| Macrosomia | 1.60 (1.21, 2.11) | 0.001 |  | 1.79 (1.08, 2.98) | 0.024 |  | 2.04 (0.56, 7.44) | 0.281 | 0.763 |
| LGA | 1.37 (1.10, 1.72) | 0.006 |  | 1.59 (1.02, 2.47) | 0.040 |  | 1.40(0.54, 3.63) | 0.488 | 0.812 |

Abbreviations: CI, confidence interval; OR, odds ratio; SGA, small-for-gestational age; LGA, large-for-gestational age.

Model 1 was adjusted for demographic characteristics including maternal age, education, employment, annual household income and study centers. Model 2 was further adjusted for drinking during pregnancy, passive smoking during pregnancy, parity, use of assisted reproductive technology, folic acid supplementation, gestational age at delivery, gestational weight gain categories. Preterm birth was not adjusted for gestational age at delivery^†^.

STable 4. Adjusted ORs (95% CIs) for pregnancy outcomes by gestational diabetes mellitus, stratified by gestational weight gain

|  | Lower | |  | Middle | |  | Higher | | *P* for interaction |
| --- | --- | --- | --- | --- | --- | --- | --- | --- | --- |
|  | OR (95% CI) | *P* value |  | OR (95% CI) | *P* value |  | OR (95% CI) | *P* value |  |
| Cesarean delivery | 1.86 (1.42, 2.44) | <0.001 |  | 1.73 (1.45, 2.07) | <0.001 |  | 1.52 (1.13, 2.03) | 0.005 | 0.943 |
| Preterm birth^†^ | 1.40 (0.95, 2.06) | 0.092 |  | 1.33 (0.99, 1.80) | 0.062 |  | 1.15 (0.64, 2.06) | 0.635 | 0.786 |
| Low birth weight | 0.73 (0.38, 1.42) | 0.357 |  | 0.91 (0.54, 1.56) | 0.739 |  | 0.73 (0.24, 2.21) | 0.573 | 0.886 |
| SGA | 1.03 (0.64, 1.65) | 0.905 |  | 0.71 (0.47, 1.07) | 0.101 |  | 0.40 (0.16, 1.02) | 0.054 | 0.308 |
| Macrosomia | 1.85 (1.01, 3.39) | 0.045 |  | 1.51 (1.08, 2.12) | 0.016 |  | 1.91 (1.28, 2.86) | 0.002 | 0.789 |
| LGA | 1.36 (0.86, 2.15) | 0.186 |  | 1.45 (1.11, 1.90) | 0.007 |  | 1.54 (1.08, 2.19) | 0.018 | 0.818 |

Abbreviations: CI, confidence interval; OR, odds ratio; SGA, small-for-gestational age; LGA, large-for-gestational age.

Model 1 was adjusted for demographic characteristics including maternal age, education, employment, annual household income and study centers. Model 2 was further adjusted for drinking during pregnancy, passive smoking during pregnancy, parity, use of assisted reproductive technology, folic acid supplementation, gestational age at delivery. Preterm birth was not adjusted for gestational age at delivery^†^.
